# Damage-Associated Molecular Patterns and Their Signaling Pathways in Primary Blast Lung Injury: New Research Progress and Future Directions

**DOI:** 10.3390/ijms21176303

**Published:** 2020-08-31

**Authors:** Ning Li, Chenhao Geng, Shike Hou, Haojun Fan, Yanhua Gong

**Affiliations:** 1 Institute of Disaster Medicine, Tianjin University, Tianjin 300072, China; lining620@tju.edu.cn (N.L.); gengchenhao@tju.edu.cn (C.G.); houshike@tju.edu.cn (S.H.); 2 Tianjin Key Laboratory of Disaster Medicine Technology, Tianjin 300072, China

**Keywords:** primary blast lung injury, ALI, DAMPs, PRRs, signaling pathways

## Abstract

Primary blast lung injury (PBLI) is a common cause of casualties in wars, terrorist attacks, and explosions. It can exist in the absence of any other outward signs of trauma, and further develop into acute lung injury (ALI) or a more severe acute respiratory distress syndrome (ARDS). The pathogenesis of PBLI at the cellular and molecular level has not been clear. Damage-associated molecular pattern (DAMP) is a general term for endogenous danger signals released by the body after injury, including intracellular protein molecules (HMGB1, histones, s100s, heat shock proteins, eCIRP, etc.), secretory protein factors (IL-1β, IL-6, IL-10, TNF-α, VEGF, complements, etc.), purines and pyrimidines and their derived degradation products (nucleic acids, ATP, ADP, UDPG, uric acid, etc.), and extracellular matrix components (hyaluronic acid, fibronectin, heparin sulfate, biglycan, etc.). DAMPs can be detected by multiple receptors including pattern recognition receptors (PRRs). The study of DAMPs and their related signaling pathways, such as the mtDNA-triggered cGAS-YAP pathway, contributes to revealing the molecular mechanism of PBLI, and provides new therapeutic targets for controlling inflammatory diseases and alleviating their symptoms. In this review, we focus on the recent progress of research on DAMPs and their signaling pathways, as well as the potential therapeutic targets and future research directions in PBLI.

## 1. Introduction

Explosions often occur in modern wars, terrorist attacks, or daily emergencies, and blast injury is a common cause of casualties [1,2]. At the moment of explosion, the explosive materials almost instantaneously convert from solid or liquid into gas. Gas expands rapidly from the explosion point and displaces the surrounding medium to create a blast overpressure (BOP), which transfers a large amount of energy to the surrounding medium to form the blast pressure wave [3]. Generally speaking, blast injuries can be divided into 4 categories, including primary (injury from direct effect of blast pressure wave), secondary (fragmentation injury), tertiary (injury caused by bodily displacement or building collapse) and quaternary (includes burn injury and inhalation of toxic substances) [3,4,5,6,7]. Furthermore, some research proposed quinary injuries, but the specific content has not reached a consensus [3,5,6,8]. In these types of injuries, primary blast injury (PBI) is a potentially life threatening, multi-system disease [4,8]. The previous common view was that blast waves mainly exert force at the air-tissue interfaces within the body. Therefore, organ systems with high air content, such as the auditory systems, pulmonary and gastrointestinal, are most likely affected by PBI [3]. Among them, lungs may be particularly susceptible to blast waves given tissue-density gradients [9]. Although recent studies indicated that blast-induced traumatic brain injury (bTBI) may be more common than previously believed [4,10], primary blast lung injury (PBLI) is still worthy of attention. The blast wave-induced acute lung injuries (ALI) are generally closed wounds rather than traditional penetrating wounds, which can be sustained in the absence of any other external signs of thoracic trauma [6]. However, it may further develop into acute respiratory distress syndrome (ARDS), a more severe physiological expression of ALI [11]. Although the current research on the pathophysiological characteristics of PBLI has been relatively clear, the mechanisms at the cellular and molecular levels are still not fully understood. Damage-associated molecular pattern (DAMP) was first proposed by Land in 2003 [12]. It is the general term for endogenous risk signals released by the organism after injury, which can trigger non-infectious and uncontrolled inflammation in the body [13,14], aggravating the lung tissue damage after an explosion [15]. This review will focus on DAMPs and their signaling pathways related to PBLI, and explore possible therapeutic targets in future clinical treatment.

## 2. Overview of Primary Blast Lung Injury

### 2.1. The Mechanism of Primary Blast Lung Injury

**Figure 1:**
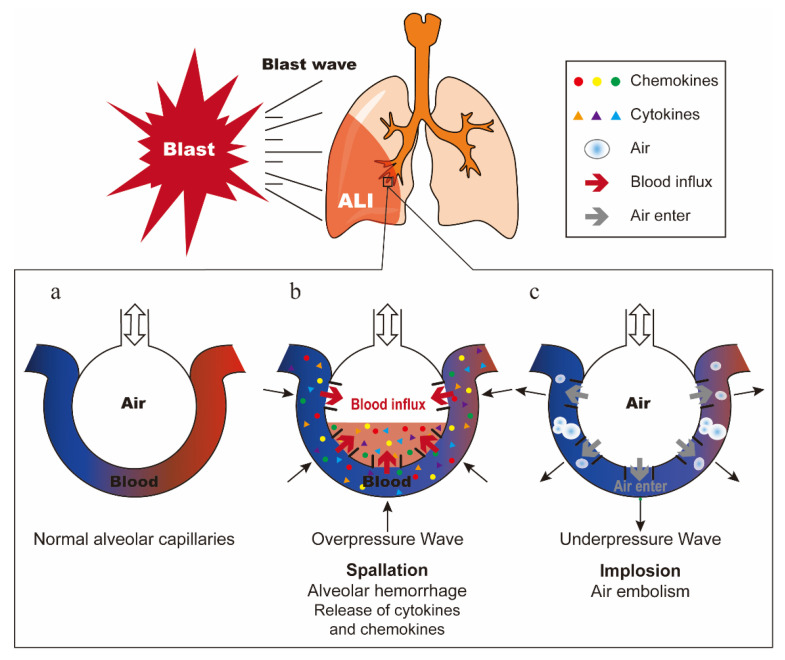
Damage mechanism of blast lung injury. (**a**) Normal alveolar capillaries. (**b**) Schematic diagram of spallation. Alveolar hemorrhage causes the release of cytokines and chemokines. (**c**) Schematic diagram of implosion. Air in the alveoli enters the pulmonary circulation, causing air embolism.

PBLI is mainly caused by the blast wave directly passing through the chest wall, which dissipates kinetic energy in the lung tissue and causes ALI [5]. In 1950, Schardin described three types of explosive forces that can cause injury: spallation, implosion, and inertia [16]. The model of normal alveolar capillaries is shown in Figure 1a. Spallation is defined as the displacement and fragmentation of the dense medium into a less dense medium (Figure 1b). Implosion is caused by the sharply reduced alveolar volume due to the high compression of air in the alveoli by overpressure wave, and then the alveoli re-expand rapidly by underpressure wave (Figure 1c). Inertia refers to the shear force produced by shock waves propagating at different speeds through different density tissues [3,17,18]. These three forces work together to cause pulmonary hemorrhage and contusion, and once the air in the alveoli enters the pulmonary circulation, it may cause air embolism, resulting in a series of symptoms such as pulmonary edema, pneumothorax, and interstitial emphysema [19,20]. The description of these three injury mechanisms is based on the blast wave generated by open air explosions. The extent to which these forces actually cause damage is not completely clear [3]. Furthermore, research shows that the reflection of blast waves in confined spaces will significantly enhance their effects and further aggravate the injury [18].

### 2.2. Pathophysiological Characteristics and Clinical Diagnosis of Primary Blast Lung Injury

The blast wave causes the rupture of alveolar capillaries, resulting in the presence of free hemoglobin (Hb) and extravasated blood in the lung tissue, induction of free radical reactions that cause oxidative damage, initiation and augmentation of a pro-inflammatory response [5,21]. Furthermore, leucocytes can be demonstrated at the hemorrhagic areas within 3 h after blast exposure, and their levels increase at 24 h or more [22]. Histological examination reveals obvious perivascular edema and massive alveolar hemorrhage within the first 12h, followed by damage to epithelial cells (at 12–24 h) and endothelial cells (at 24–56 h) [23].

Clinically, patients with PBLI often have dyspnea, shallow and frequent breathing [24]. In chest imaging, PBLI appears as ground-glass opacity (GGO), consolidation, and bilateral fluffy infiltrates that resemble a “butterfly” or “bat wing” [4,25]. In addition, alveolar hemorrhage, laceration of lung parenchyma, subcutaneous emphysema, pneumothorax, and hemothorax can also be observed [25,26].

### 2.3. The Medical Treatment of Primary Blast Lung Injury

After an explosion incident, the emergency personnel should first identify whether there is the risk of PBLI according to the place where the explosion occurred and the distance between the wounded and the center of explosion [5]. It is imperative to perform pre-hospital first aid, and notify the relevant department staff in advance to make relevant preparations, so as to ensure the smooth progress of follow-up treatment [4]. The current in-hospital treatment strategies for PBLI mainly include fluid resuscitation, hyperbaric oxygen therapy, mechanical ventilation, etc. [27,28,29], and can be combined with drugs that inhibit inflammatory factors, scavenge oxygen free radicals, relieve smooth muscle spasm, and improve lung ventilation [30]. These interventions aim to control the development of the disease and maintain the vital signs of patients. In addition, the application of new biotechnology such as mesenchymal stem cells (MSCs) in the treatment of PBLI has become a research hotspot in recent years [31,32]. However, due to insufficient research on the molecular mechanism of PBLI, there is no specific medicine for the treatment of PBLI. Therefore, the study of DAMPs in PBLI is of great significance for exploring potential therapeutic targets and developing targeted treatment methods.

## 3. DAMPs and Their Sensing Receptors

### 3.1. Damage-Associated Molecular Pattern

The immune system defends our body with the mechanism of “self versus non-self” recognition. However, it can be activated not only by exogenous “non-self” substances, but also by endogenous DAMPs [33]. DAMPs are numerous intracellular molecules with physiological functions, which exist in the nucleus, mitochondria, or cytoplasm [34,35,36]. Under normal physiological conditions, DAMPs cannot be recognized by the immune system, but they are released when cells die or are subjected to stress [37,38].

When released into the extracellular environment, DAMPs can activate innate immune cells, such as polymorphonuclear neutrophils (PMNs), natural killer cells (NKs), macrophages, and dendritic cells (DCs), as well as non-immune cells, such as epithelial cells, endothelial cells, and fibroblasts [39]. The activation of these cells results in the release of various cytokines and chemokines, which further recruit inflammatory cells and activate adaptive immune responses. In addition, some DAMPs can directly activate adaptive immune cells to generate immune responses. Whereas the local inflammatory response plays an important role in tissue repair and regeneration, excessive or persistent inflammation may also lead to a systemic and uncontrolled inflammatory response inducing remote organ failure [40].

### 3.2. DAMP-Sensing Receptors

**Figure 2:**
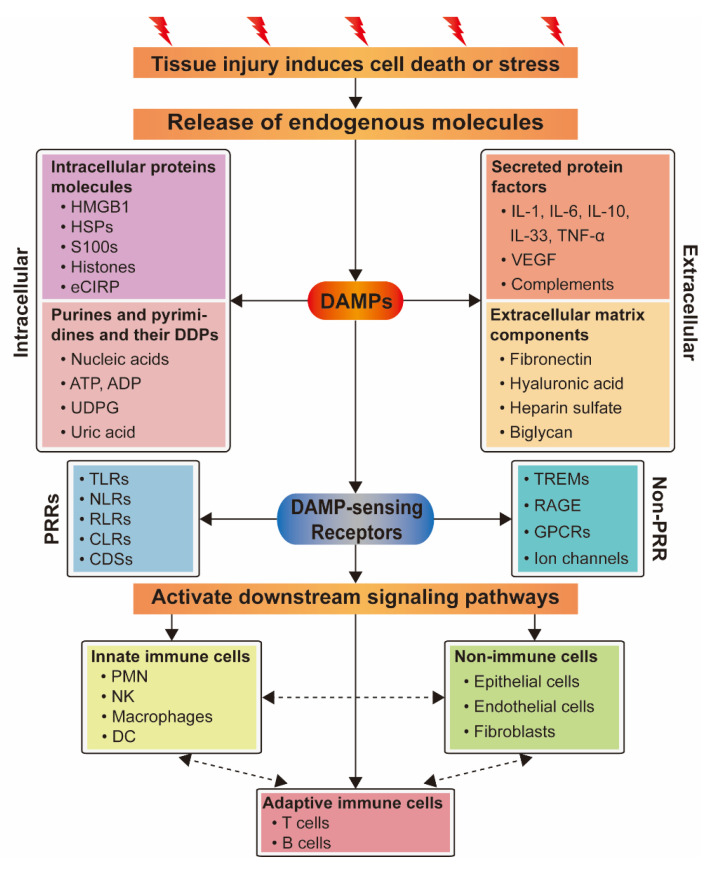
Relationship between DAMPs and their sensing receptors and the activation of different types of cells. DAMPs are released from cells which are dead or stressed due to tissue injury, and include intracellular protein molecules, secreted protein factors, purine and pyrimidine molecules, and their derived degradation products (DDPs) and extracellular matrix components. They activate downstream signaling pathways by recognizing their sensing receptors to activate innate immune cells or non-immune cells, then adaptive immune cells, and also directly activate adaptive immune cells to initiate inflammatory response. Different cellular populations communicate with each other through cytokines and chemokines.

DAMPs released by injured organs, tissues, or cells can be detected by multiple receptors including pattern recognition receptors (PRRs) (Figure 2), which can, in turn, activate multiple pathways to regulate the inflammatory response. Moreover, one DAMP may bind to a variety of receptors and participate in different pathways [40].

#### 3.2.1. Pattern Recognition Receptors Bound by Damage-Associated Molecular Patterns

PRRs are expressed by innate immune cells such as PMNs, NKs, macrophages, and DCs, and mainly include toll-like receptors (TLRs), NOD-like receptors (NLRs), retinoic acid-inducible gene-I (RIG-I)-like receptors (RLRs), C-type lectin receptors (CLRs), and multiple cytoplasmic DNA sensors (CDSs) [41]. TLRs recognize DAMP molecules and participate in inflammatory response [42]. NLRs promote the activation of inflammatory bodies [43]. RLRs are one of the main receptor families for sensing viral RNA [44]. The main function of CLRs is to induce pro-inflammatory response [45]. CDSs play essential roles in the development of inflammatory diseases and tumors [46].

1. Toll-like receptors

TLRs are located on the cell surface or inside the cell, and can recognize DAMPs such as nucleic acids, HMGB1, heat shock proteins (HSPs), and S100 proteins. Most TLRs (except TLR3) are recruited by myeloid differentiation protein 88 (MyD88) -dependent IL-1 receptor-associated kinase (IRAK), which then activates nuclear factor-κB (NF-κB) to promote inflammatory response by upregulating the expression of tumor necrosis factor-α (TNF-α), interleukin 6 (IL-6), and IL-8 [40,47]. TLR3 participates in the inflammatory response through TRIF dependent pathway [42].

2. NOD-like receptors

NLRs are PRRs located in the cytoplasm, which can recruit pro-caspase-1 to start the assembly of inflammatory bodies after being activated by DAMPs such as ATP and oxidized mitochondrial DNA (ox-mtDNA), thereby inducing apoptosis and secretion of IL-1β, IL-18 and other cytokines through the activation of caspase-1 [43,48].

3. Retinoic acid-inducible gene-I (RIG-I)-like receptors

The RLR family includes RIG-I, MDA5, and LGP2, which can distinguish between exogenous RNA and endogenous RNA to resist pathogens [44]. It can sense DAMPs such as endogenous 5′ppp RNA and unedited long self-dsRNA in vivo, and promote the production of type I interferon (IFN-I) and other cytokines and chemokines [49,50].

4. C-type lectin receptors

Some members of the CLR family, such as macrophage-inducible C-type lectin (MINCLE) and dendritic cell natural killer lectin group receptor 1 (DNGR1), are related to the development of inflammation diseases after binding to DAMPs. DNGR1 is a DC receptor which can be activated by F-actin to downregulate the production of the anti-inflammatory cytokine IL-10 in DCs and aggravate the inflammatory response [51]. MINCLE binds to SAP130 protein to induce pro-inflammatory response [45].

5. Cytoplasmic DNA sensors

Cyclic GMP–AMP synthase (cGAS) and absent in melanoma 2 (AIM2) are two important DNA sensors in the cytoplasm, which play essential roles in the development of inflammatory diseases and tumors [52,53]. Some studies have shown that self-DNA, produced by immunogenic cell death or nuclear DNA damage, can activate AIM2 or cGAS and promote the production of IFN-I and other cytokines and chemokines [46].

#### 3.2.2. Other Receptors/Channels Bound by DAMPs

In addition to PRRs, DAMPs can activate receptors including triggering receptors expressed on myeloid cells (TREMs), receptor for advanced glycation end products (RAGE), several G-protein-coupled receptors (GPCRs), and ion channels [41]. TREM activation causes cytokine secretion, leading to inflammation response. The interaction between 35 kDa transmembrane receptor RAGE and its ligands also leads to the activation of pro-inflammatory genes [54,55]. GPCRs are known as seven-(pass)-transmembrane receptors and involved in many diseases via the cAMP or the phosphatidylinositol signal pathway [56]. Ion channels trigger the production of NLRP3 inflammasome and participate in the inflammatory response [57].

1. Triggering receptors expressed on myeloid cells

TREM1 and TREM2 are cell surface innate immune receptors which act as members of the immunoglobulin variable (IgV) domain receptor superfamily [58]. TREM1 is expressed on myeloid cells, epithelial cells, endothelial cells, and fibroblasts, and interacts with DAMPs such as HMGB1, HSP70, and actin to promote the secretion of pro-inflammatory cytokines and chemokines [59,60]. TREM2 is highly expressed in myeloid cells such as DCs, monocytes, and macrophages, and binds to DAMPs such as HSP60, apolipoproteins (APOJ and APOE), and low-density lipoprotein (LDL) to regulate cell differentiation, phagocytosis, chemotaxis, and other processes, and participate in inflammatory diseases [61,62].

2. Receptor for advanced glycation end products

RAGE is a transmembrane protein that belongs to the immunoglobulin receptor superfamily and is expressed by multiple cell types, including neutrophils, monocytes, smooth muscle cells, endothelial cells, and cancer cells, and mediates cell response to DAMPs such as HMGB1, S100 proteins, advanced glycation end products (AGEs), and DNA to promote the expression of pro-inflammatory genes, as well as cell migration, proliferation, and apoptosis [63].

3. G-protein-coupled receptors

Various DAMPs can also promote inflammation via GPCRs. The N-formyl peptide receptors (FPRs) can recognize endogenous N-formylated peptides and promote the chemotaxis of neutrophils and monocytes/macrophages [64]. P2Y receptors (P2YRs) can sense extracellular nucleotides and promote the migration and activation of multifarious immune cells [65]. Calcium-sensing receptors (CaSRs) and G-protein-coupled receptor family C group 6 member A (GPRC6A) can recognize extracellular Ca^2+^, promote monocyte/macrophage recruitment and the activation of the NLRP3 inflammasome [66].

4. Ion channels

Transient receptor potential (TRP) channels and P2X receptors are two types of ligand-gated ion channels, which can promote the production of cytokines and chemokines and the activation of NLRP3 inflammasome by recognizing mitochondria-derived reactive oxygen species (ROS) and ATP, respectively, and participate in the inflammatory response [57,67].

### 3.3. Crosstalk Between DAMP and Their Sensing Receptors

In summary, DAMPs can bind to related receptors or channel proteins and activate downstream signaling pathways, leading to large-scale cytokine release, including IL-1, IL-6, IL-8, IL-12, TNF-α, and type I/type II IFN. These mediators enhance the activation, maturation, proliferation, and recruitment of immune cells at the injured regions, leading to the indirect activation of innate and adaptive immune cells (such as DCs or T cells) (Figure 2).

## 4. DAMPs and Their Signaling Pathways Related to PBLI

### 4.1. Intracellular Protein Molecules

#### 4.1.1. Nuclear Protein HMGB1

**Figure 3:**
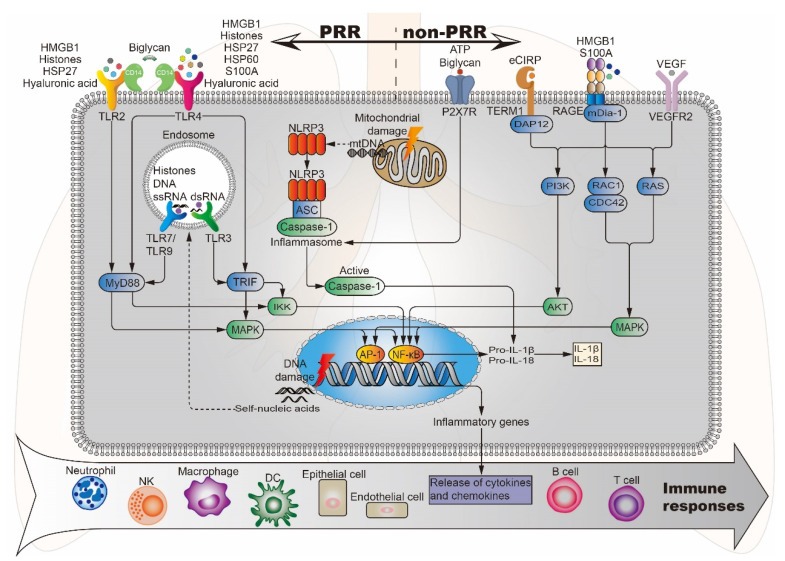
Potential DAMPs and related signaling pathways in BLI. Lung injury-related DAMPs can activate PRR (TLR2/3/4/7/9, NLRP3) and non-PRR (P2X7R, RAGE, TERM1, VEGFR2) to activate the corresponding signaling pathways, induce the release of cytokines and chemokines, and further stimulate effector cells to produce an immune response. DAP12, DNAX activation protein 12; mDia-1, mammalian diaphanous-related formin 1; ASC, apoptosis-associated speck-like protein.

HMGB1 is a late inflammatory mediator associated with sepsis, malignancy, and immune disease, and is one of the most intensively studied DAMPs. Levels of HMGB1 may reflect the severity of inflammation and tissue damage, indicating a potential role for HMGB1 as a biomarker of ALI, and a potential target for blocking inflammatory pathways [68]. Under normal physiological conditions, HMGB1 is located in the nucleus, but it is passively released from necrotic cells after PBLI. HMGB1 triggers signaling cascades by combining with RAGE, TLR2, and TLR4, thereby inducing the activation of the NF-κB pathway and participating in the inflammatory response [14] (Figure 3). Li et al. [69] demonstrated that HMGB1 participates in ALI by activating double-stranded RNA-dependent protein kinase (PKR) in macrophages and inducing macrophage (M1) polarization via TLR2- and TLR4-mediated NF-κB signaling pathways in a mouse model of ALI induced by the bacterial endotoxin lipopolysaccharide (LPS). In addition, HMGB1 levels are also related to processes such as hyperfibrinolysis and complement activation and participate in systemic inflammatory responses [70].

#### 4.1.2. Histones

Histones are nucleoproteins involved in the compaction of DNA into nucleosomes. They provide structural stability to chromatin and regulate gene expression within the nucleus. However, once released from the nucleus into the cytoplasm or body fluids, histones can act as a DAMP, activating the immune system and causing further cytotoxicity. They interact with TLRs, complement and cell membrane phospholipids inducing endothelial and epithelial cytotoxicity, TLR2/TLR4/TLR9 activation and pro-inflammatory cytokine/chemokine release via MyD88, NF-κB and NLRP3 inflammasome-dependent pathways [71] (Figure 3). Abrams et al. [72] correlated circulating histone levels in patients with severe trauma with respiratory failure and sequential organ failure assessment (SOFA) scores, and finally investigated their cause-effect relationship using cellular and mouse models. The results showed that circulating histones surged immediately after trauma to levels that were toxic to cultured endothelial cells and acted as mediators of damage to the distal organs. Among distal organs, the lung is predominantly affected. The toxicity of histones stems from their affinity for phosphate groups within DNA and phospholipids. The interaction with phospholipids leads them to integrate into the cell membrane and cause large inward ion currents and calcium influx. These damaging effects lead to endothelial damage, coagulation activation, cytokine release, and neutrophil extracellular trap (NET) formation. Based on the above study, circulating histones may be candidate therapeutic targets for improving survival outcomes in trauma-associated lung injury patients.

#### 4.1.3. Calcium Binding Protein S100A

The S100 protein family consists of 25 members [73], which exert multiple intracellular and extracellular functions. Within the cells, S100 proteins regulate processes such as cell proliferation, differentiation, migration, energy metabolism, Ca2+ homeostasis, inflammation, and cell death [74]. When tissue damage occurs, certain S100 proteins are released into the extracellular space to act as DAMPs through the interaction with TLR4 and RAGE to participate in inflammation [75] (Figure 3). S100A8 and S100A9 are myeloid cell-derived proteins that can form homodimers and heterodimers. Studies have shown that the systemic and local levels of S100A8 and S100A9 are increased in several inflammatory lung diseases [76]. Chakraborty et al. [77] found that S100A8/A9 is a necessary factor for neutrophil recruitment to lungs in S100-mediated activation of alveolar epithelial cells (AECs). S100A8 promotes ALI via TLR4-dependent activation of AECs to produce inflammatory factors/chemokines and then enhance inflammatory responses. Therefore, blocking the interaction between S100A8 and TLR4 by targeting S100A8 residues involved in TLR4 binding could represent a new option for the treatment of ALI and other inflammatory lung diseases.

#### 4.1.4. Heat Shock Proteins

HSPs are molecular chaperones with the function of controlling intracellular transport and preventing misfolding of polypeptide chains. These proteins are named by their molecular weight [40]. The expression of HSPs is at low levels under normal physiological conditions. However, during times of physiological stress, the expression of HSPs increases and HSPs can be released from necrotic cells into the extracellular compartment, which promotes inflammation via the induction of multiple pro-inflammatory cytokines [78]. For example, exogenous HSP27 activates the NF-κB pathway through TLR2 and TLR4, upregulating the expression of monocyte chemoattractant protein (MCP)-1, intercellular adhesion molecule (ICAM)-1, IL-6, and IL-8 in human coronary vascular endothelial cells (HCVECs) [79]. HSP60 activates NF-κB through TLR4 and promotes the production of various pro-inflammatory and neurotoxic factors in mouse BV2 microglia [80] (Figure 3).

The role of HSPs in the inflammatory response caused by PBLI is unclear, but some HSPs also show protective effects on lung injury. Wong et al. [81] overexpressed HSP70 gene in a cell model of hyperoxia-induced lung injury, which has a protective effect on A549 cells, suggesting that HS70 can protect human respiratory epithelial cells from injury under a high oxygen environment. Tanaka et al. [82] found that HSP70 can prevent bleomycin-induced inflammation and lung injury. Therefore, the application of HSP70 in the treatment of PBLI is expected to become a new research direction.

#### 4.1.5. Cold-Inducible RNA-Binding Protein

CIRP is an evolutionarily conserved RNA partner [83]. Under normal physiological conditions, CIRP is synthesized in the cytoplasm and then returned to the nucleus, where it regulates RNA transcription and processing, and also regulates the translation and transport of mRNA in the cytoplasm. However, during hypoxia and inflammation, CIRP transfers from the nucleus to the cytosol and is gradually released into the extracellular space. Extracellular CIRP (eCIRP) can act as a DAMP to induce inflammatory responses in dendritic cells, lymphocytes, macrophages, and neutrophils [84]. Gurien et al. [85] discovered that TREM-1 serves as a new receptor of eCIRP to exaggerate inflammation in a mouse model of hemorrhagic shock (HS)-induced ALI (Figure 3). Use of a novel short peptide derived from human eCIRP known as M3 to inhibit the interaction between eCIRP and TREM-1 significantly reduced the expression of pro-inflammatory cytokines chemokines MIP-2, TNF-α, interleukin 1β (IL-1β), IL-6, and KC in the lungs of HS mice and decreased lung injury score. This study suggests that blocking the interaction between eCIRP and TREM-1 can be used as a new approach to reduce inflammation in ALI.

### 4.2. Secretory Protein Factors

#### 4.2.1. Cytokines IL-1, IL-6, IL-10, IL-33, and TNF-α

Cytokines are small messenger molecules that are produced, activated, and released under trauma. IL-1β is a member of the interleukin 1 cytokine family, that is produced by activated macrophages. As a highly essential upstream pro-inflammatory cytokine, IL-1β activates downstream inflammatory factors such as TNF-α via IL-1 receptors to impinge on various cellular functions such as cell proliferation, differentiation, and apoptosis [86]. IL-6 is derived from monocytes/macrophages, endothelial cells, fibroblasts, and smooth muscle cells [87], acting as both a pro-inflammatory cytokine and an anti-inflammatory myokine [88]. IL-6 can be secreted by macrophages in response to specific microbial molecules, which in turn induces intracellular signal transduction cascades, thereby causing the production of inflammatory cytokines. The role of IL-6 as an anti-inflammatory myokine is mediated by its inhibition of TNF-α and IL-1, and activation of IL-1 receptor antagonist (IL-1ra) and IL-10 [89]. IL-10, also known as human cytokine synthesis inhibitory factor (CSIF), performs signal transduction by binding a tetrameric complex formed by two IL-10 receptor 1 (IL-10R1) and two IL-10R2 subunits [90]. IL-10 is a multifunctional cytokine expressed in different types of cells, which is recognized as an anti-inflammatory and immunosuppressive factor. It can downregulate the expression of Th1 cytokines, MHC-II antigens, and costimulatory molecules on macrophages [91]. TNF-α is primarily produced and secreted by activated monocytes/macrophages and is an important medium and starting point in the inflammatory response [92]. IL-1β, IL-6, and TNF-α are pro-inflammatory cytokines, while IL-10 is an anti-inflammatory cytokine. Tong et al. [93] found that the mRNA and protein expression of IL-1 β, IL-6, and TNF-α increased, while the mRNA and protein expression of IL-10 decreased after PBLI, indicating the occurrence of inflammation. IL-1β, IL-6, IL-10, and TNF-α are extensively used as biomarkers to assess the degree of inflammatory response and are indicators of whether the treatment method has anti-inflammatory effects. Zhang et al. [94] studied the therapeutic effect of perfluorocarbon (PFC) on PBLI at the cellular level. The results showed that PFC significantly inhibited the expression of IL-1β, IL-6, and TNF-α in the supernatant of A549 cells achieving anti-inflammatory effects. The potential mechanism may be associated with the inhibition of the NF-κB and MAPK signaling pathways to reduce the inflammatory response.

In addition, targeting cytokine-related signaling pathways, inhibiting the release of pro-inflammatory factors, or increasing the level of anti-inflammatory factors is a new method of treatment for cytokine storms caused by ALI. In coronavirus disease 2019 (COVID-19)-induced ALI, a large number of patients develop cytokine storm, also known as cytokine release syndrome (CRS), suggesting that it may be the main pathological cause of COVID-19 [95]. IL-6 is a key molecule of CRS. Blocking the IL-6 signaling pathway with IL-6R antibody can inhibit CRS, thus the IL-6R antagonist Tocilizumab may be key to reducing the mortality of CRS of severe COVID-19 [96]. The application of IL-6R antagonists to block the IL-6 signaling pathway also provides new ideas for the pharmacological treatment of PBLI.

#### 4.2.2. Vascular Endothelial Growth Factor

Under physiological conditions, VEGF can promote the formation and development of vascular tissue. However, during the pathologic process, VEGF can change the permeability of blood vessels, increase inflammatory exudation, and aggravate the inflammatory response. VEGF gene family members include VEGF-A, VEGF-B, VEGF-C, VEGF-D, and placental growth factor (PLGF) [97]. Among them, the main mediator of angiogenesis is the dimeric glycoprotein VEGF-A, which is usually referred to as VEGF [98,99]. In adults, the lung is the major source of VEGF, where VEGF and its receptor (VEGFR) are largely expressed in type II alveolar epithelial cells, activated macrophages, and respiratory epithelial cells in lung tissue [100,101]. After PBLI, the alveolar epithelium and alveolar capillaries are exposed to high concentrations of VEGF, which increase the vascular permeability, resulting in an increased inflammatory response during the exudation period and exacerbating the condition of PBLI [102]. Qin et al. [103] found that in the ARDS model, levels of TNF-α, IL-6, and VEGF in bronchoalveolar lavage fluid (BALF) were significantly higher than those of the control group, resulting in an imbalance between pro-inflammatory and anti-inflammatory responses. In addition, Lin et al. [98] suggested that VEGF participates in fat embolism (FE)-induced ALI via VEGFR-2 and MAPK cascade, which induce IL-1β release and inducible nitric oxide synthase (iNOS) upregulation (Figure 3). Treatment with VEGFR-2 antagonist SU-1498 significantly attenuated the inflammatory response and histological damage. Therefore, whether inhibitors targeting the VEGF signaling pathway can be applied in the treatment of PBLI may be a potential new research direction.

#### 4.2.3. Complements

The complement system is an integral part of the innate immune system and participates in the important early inflammatory processes, which can lead to multiple organ dysfunction after trauma. A series of studies have shown that activation of the complement system plays a key role in the pathogenesis of ALI [104]. Yang et al. [105] detected the complement activation, plasminogen, and myeloperoxidase levels by complement hemolytic assay (CH50) and/or ELISA in a rat model of blast-induced multiple organ failure (MOF) from a compressed air-driven shock tube. The results showed that the level of complement components C3 and C1q in the blood significantly decreased at 1 and 3 h after blast, indicating that complement activation occurred. Plasminogen levels also dropped significantly at 1 and 3 h after blast, indicating that plasminogen was converted to plasmin, resulting in fibrinolysis. In addition, the study also showed that systemic inflammation occurred at 24 h after injury. Therefore, it can be inferred that targeting the complement and fibrinolytic systems can serve as a new therapy for PBLI. In a rat model of PBLI induced by a blast wave from a compressed air-driven shock tube, Li et al. [106] indicated that early administration of decay-accelerating factor (DAF) efficiently inhibited systemic and local inflammation, and mitigated blast-induced lung injury. Its underlying mechanism might be regulated by the C3a-C3aR-HMGB1-transcriptional factor axis, suggesting that complement and HMGB1 may be potential therapeutic targets for ameliorating ALI after blast injury.

### 4.3. Purines and Pyrimidines and Their Derived Degradation Products

#### Nucleic Acids

**Figure 4:**
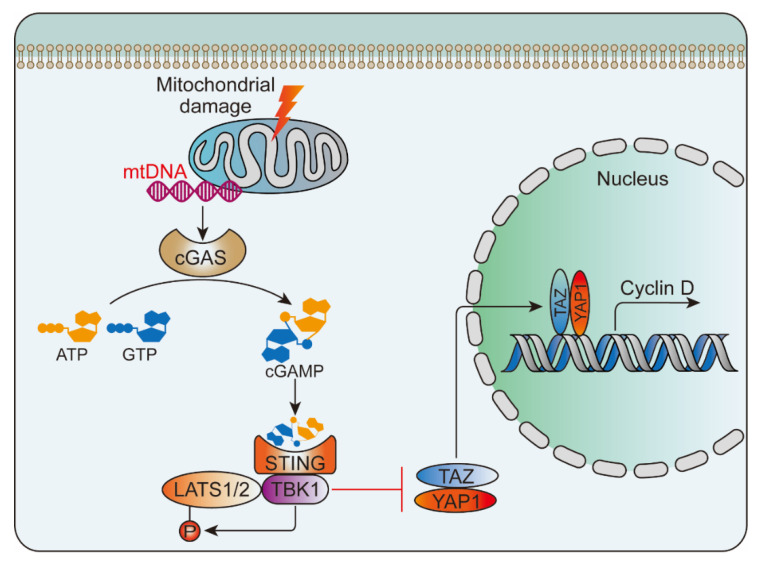
mtDNA-triggered cGAS-YAP pathway. mtDNA was released from the damaged mitochondria and then activated the cGAS-STING signaling pathway, which inhibited the nuclear translocation of YAP1 and inhibited cyclin D mediated endothelial proliferation. STING, stimulator of interferon genes; TBK1, TANK-binding kinase 1; LATS1/2, large tumor suppressor kinase 1/2; TAZ, transcriptional coactivator with PDZ-binding motif.4.3.2. Adenosine triphosphate.

Following tissue damage, nucleic acids in the nucleus and mitochondria are released into the cytoplasm and blood. Once leaving their original subcellular location, these nucleic acids may be modified and recognized as foreign, eliciting an innate immune response. The discovered nucleic acid ligands are TLR9 (recognition of DNA), TLR7 (recognition of single-stranded RNA) and TLR3 (recognition of double-stranded RNA). Their immune complexes can directly activate plasmacytoid pre-dendritic cells, resulting in the production of interferon-alpha (IFN-α) and initiation of inflammation [78] (Figure 3). Wu et al. [107] studied the activation of NLRP3 inflammasome by intratracheal administration of mitochondrial DNA (mtDNA) in mouse. The results showed that extracellular mtDNA promotes NLRP3 inflammasome activation, acute pulmonary inflammation, and injury through TLR9, p38 MAPK, and NF-κB pathways. Huang et al. [108] demonstrated that LPS treatment resulted in activation of the pore-forming protein gasdermin D, which formed mitochondrial pores and induced mtDNA release into the cytosol of endothelial cells. Yes-associated protein (YAP) is one of the most important transcriptional coactivators of the Hippo pathway [109], and participates in regulating cell proliferation, survival, and migration [108,110]. mtDNA was recognized by the DNA sensor cGAS and generated the second messenger cyclic GMP-AMP (cGAMP), thereby inhibiting endothelial cell proliferation by down-regulating the YAP1 signal (Figure 4) [108]. This indicated that the regenerative capacity of surviving endothelial cells in inflammatory injury was compromised. In an experimental model of inflammatory lung injury, deletion of cGAS in mice restored the regeneration of the endothelium. It is suggested that the cGAS-YAP signaling pathway activated by gastermin D could serve as a potential strategy for restoring endothelial function after inflammatory injury [108]. The release of mtDNA induced by mitochondrial damage also exists after PBLI, and its mechanism still needs to be confirmed by further studies.

Another DAMP originating from mitochondria is ATP. This nucleotide can be released from apoptotic or necrotic cells after tissue damage, contributing to the induction of inflammation by activation and recruitment of various inflammatory cells, such as macrophages, neutrophils, and DCs [33]. ATP is a potent activator of NLRP3 inflammasome. As a result of its activation, DAMPs such as IL-1, IL-18, HMGB1, and S100A9 are released from hematopoietic stem/progenitor cells (HSPCs) (Figure 3). These DAMPs are vital activators of the complement cascade (ComC) in the mannan-binding lectin (MBL)-dependent pathway [111].

Cicko et al. [112] examined the role of extracellular ATP in recruiting inflammatory cells to the lung in a mouse model of LPS-induced ALI. The results showed that intratracheal application of LPS caused acute accumulation of ATP in the BALF and lungs of mice. Inhibition of P2X7 receptor (P2X7R) signaling by a specific antagonist and knock-out experiments can reduce ATP level, neutrophil number, and pro-inflammatory cytokine concentration in BALF, thereby improving the inflammatory response. Further research indicated that the expression of P2X7R in immune cells was responsible for ATP-induced inflammatory response bursts, suggesting that P2X7R may be a new therapeutic target for ALI/ARDS.

### 4.4. Extracellular Matrix Components

Extracellular matrix components (ECMs) are macromolecular substances that exist on the surface of cells and between cells, and include fibronectin, hyaluronic acid, heparan sulfate, biglycan (BGN), etc. These substances form a network structure, which maintains the morphology of the cell while regulating the physiological activity of the cell. When tissues are inflamed, ischemic, hypoxic, or injured, the ECMs are degraded by proteases released by injured or necrotic cells. Some degraded ECMs have the biological activity of DAMPs and can be combined with different types of PRRs to regulate the inflammatory response and participate in the repair of tissue damage [113]. Hyaluronic acid acts as an important component of extracellular matrix, and has a significant role in phases of skin wound repair, such as tissue regeneration, angiogenesis, and inflammation response [114]. Biglycan has the function of reducing the growth rate and bone mass of mice [115]. Recently research also found that biglycan may be a molecular switch between inflammation and autophagy [116].

#### 4.4.1. Hyaluronic Acid

Previous studies have shown that the glycosaminoglycan hyaluronic acid (HA) produced by injured tissues with impaired clearance can be recognized as a DAMP signal to activate lung innate immunity [117]. Jiang et al. [118] reported that low molecular weight hyaluronic acid (LMW-HA) increased the expression of CXCL2, MIP-2, and TNF-α in cells by activating TLR2 and TLR4 on the surface of macrophages in a mouse model of bleomycin-induced lung injury (Figure 3). Although the number of inflammatory cells in the BALF of the mouse lung injury model was significantly decreased after blocking the interaction of HA with TLR2 and TLR4 with antibodies, the HE staining of lung tissue showed that the diffuse alveolar damage caused by inflammation was significantly aggravated. The researchers further found that the overexpression of high molecular weight hyaluronic acid (HMW-HA) in vivo can significantly reduce the mortality of ALI mice caused by bleomycin. The mechanism may be that hyaluronic acid activates the NF-κB pathway through TLR, which plays an anti-apoptotic role [78]. It can be seen that HMW-HA and LMW-HA play different roles in ALI. Xu et al. [119] also confirmed the protective effect of high molecular weight hyaluronan on fine particulate matter-induced ALI. This process is achieved by inhibiting ROS-apoptosis signal-regulating kinase-1 (ASK1)-p38/c-Jun N-terminal kinase (JNK) pathway-mediated apoptosis of epithelial cells. Therefore, the application of HMW-HA in PBLI may be a promising treatment strategy, but the treatment of the inflammatory response induced by LMW-HA as a DAMP needs further exploration.

#### 4.4.2. Biglycan

BGN is a ubiquitous small leucine-rich proteoglycan in the extracellular matrix, which can act as a DAMP to interact with PRRs such as TLRs, and participate in the inflammatory response by generating pro-inflammatory mediators and recruiting leukocytes to the injury site [36]. Koslowski et al. [120] found BGN deposited in the lung tissue of rat in bleomycin-induced lung injury. Babelova et al. [121] found that BGN interacted with TLR2/4 and P2X4/P2X7 receptors in macrophages, inducing the formation of NLRP3/ASC inflammasome and activating caspase-1 to secrete mature IL-1β to promote inflammatory response (Figure 3). Studies based on biglycan knock-out (Bgn-/0) mice found that biglycan deficiency in the lung, a major target organ in sepsis, resulted in attenuation of IL-1β mRNA expression, reducing the inflammatory response. The latest research on the mechanism of action of BGN as a DAMP in inflammation is mostly conducted in the context of kidney injury. Roedig et al. [122] found in a mouse model of renal ischemia/reperfusion injury (IRI) that BGN is a novel high-affinity ligand for CD14 in macrophages, which can evoke the activation of p38, p44/42 MAPKs, and p65 NF-κB pathways in a CD14-dependent manner to regulate inflammatory response. Meanwhile, Poluzzi et al. [123] found that the biglycan-CD44 interaction increased M1 macrophage autophagy and the number of renal M2 macrophages and reduced tubular damage following IRI. This process is realized by CD44/TLR4 signaling axis. Subsequently, Roedig et al. [116] discussed the mechanism of BGN as a molecular switch, triggering signals in both directions of promoting inflammation and autophagy. Therefore, it is necessary to selectively develop novel therapeutic regimens targeting the interactions between biglycan, TLRs, coreceptors, and adapter molecules. Based on the above research, BGN may be a potential therapeutic target for inflammatory response after PBLI.

## 5. Conclusions

PBLI is essentially an acute lung injury caused by overpressure mechanical force. After PBLI, DAMPs are released from the necrotic or stressed cells to recruit the effector cells, which release cytokines, and then enter the acute exudative phase of ALI. With the release of inflammatory cytokines (TNF-α, IL-1β, etc.) and chemokines (CXC, CC, CX3C, etc.) and with the generous activation of inflammatory leukocytes, alveolar capillary membrane damage and type I alveolar epithelial cell necrosis appear and inflammatory exudates gather in the lung stroma, resulting in acute pulmonary edema and acute respiratory failure, which can further develop into more serious ARDS [78]. DAMPs are “danger” signals emitted by the body after injury and trigger inflammatory responses, implicated in local tissue repair. However, the imbalance of inflammatory response can cause pathological inflammation and related diseases. Although the research on DAMPs and their signaling pathways in the field of lung injury-related inflammatory response has been comprehensive, the research in the field of PBLI is still in its infancy. There is still a lot of work to verify the role of existing DAMPs in PBLI, such as ECM components, HSPs, nucleic acids, ATP, etc. It is also necessary to explore and discover new unique DAMPs relevant to PBLI. In the future, the in-depth interpretation of the molecular mechanism of DAMPs and their downstream signaling pathways in PBLI can provide new treatment avenues and targets for controlling the inflammatory response and alleviating the patient’s condition after lung injury caused by blast waves.

## Abbreviations

AECs	Alveolar Epithelial Cells
AGEs	Advanced Glycation End Products
AIM2	Absent in Melanoma 2
ALI	Acute Lung Injuries
ARDS	Acute Respiratory Distress Syndrome
ASC	Apoptosis-Associated Speck-Like Protein
ASK1	Apoptosis Signal-Regulating Kinase-1
ATP	Adenosine Triphosphate
BALF	Bronchoalveolar Lavage Fluid
BGN	Biglycan
BOP	Blast Overpressure
bTBI	Blast-Induced Traumatic Brain Injury
CaSRs	Calcium-Sensing Receptors
CDSs	Cytoplasmic DNA Sensors
cGAMP	Cyclic GMP-AMP
cGAS	GMP–AMP Synthase
CLRs	C-Type Lectin Receptors
ComC	Complement Cascade
COVID-19	Coronavirus Disease 2019
CRS	Cytokine Release Syndrome
CSIF	Cytokine Synthesis Inhibitory Factor
DAF	Decay-Accelerating Factor
DAMP	Damage-Associated Molecular Pattern
DCs	Dendritic Cells
DDPs	Derived Degradation Products
DNGR1	Dendritic Cell Natural Killer Lectin Group Receptor 1
eCIRP	Extracellular Cold-Inducible RNA-Binding Protein
ECM	Extracellular Matrix
FE	Fat Embolism
FPRs	N-Formyl Peptide Receptors
GGO	Ground-Glass Opacity
GPCRs	G-Protein-Coupled Receptors
GPRC6A	G-Protein-Coupled Receptor Family C Group 6 Member A
HA	Hyaluronic Acid
Hb	Hemoglobin
HCVECs	Human Coronary Vascular Endothelial Cells
HMGB1	High Mobility Group Box 1
HMW-HA	High Molecular Weight Hyaluronic Acid
HSPs	Heat Shock Proteins
ICAM	Intercellular Adhesion Molecule
IFN-I	Type I Interferon
IFN-α	Interferon-Alpha
IgV	Immunoglobulin Variable
IL-10R1	IL-10 Receptor 1
IL-1ra	IL-1 receptor antagonist
IL-6	Interleukin 6
IL-6R	Interleukin-6 Receptor
iNOS	Inducible Nitric Oxide Synthase
IRAK	IL-1 Receptor-Associated Kinase
IRI	Ischemia/Reperfusion Injury
JNK	C-Jun N-Terminal Kinase
LATS1/2	Large Tumor Suppressor Kinase 1/2
LDL	Low-Density Lipoprotein
LMW-HA	Low Molecular Weight Hyaluronic Acid
LPS	Lipopolysaccharide
MBL	Mannan-Binding Lectin
MCP	Monocyte Chemoattractant Protein
MINCLE	Macrophage-Inducible C-type Lectin
MOF	Multiple Organ Failure
MSCs	Mesenchymal Stem Cells
mtDNA	Mitochondrial DNA
MyD88	Myeloid Differentiation Protein 88
NET	Neutrophil Extracellular Trap
NF-kB	Nuclear Factor-κB
NKs	Natural Killer Cells
NLRs	NOD-Like Receptors
ox-mtDNA	Oxidized Mitochondrial DNA
P2X7R	P2X7 Receptor
P2YRs	P2Y Receptors
PBI	Primary Blast Injury
PBLI	Primary Blast Lung Injury
PFC	Perfluorocarbon
PKR	Double-Stranded RNA-Dependent Protein Kinase
PLGF	Placental Growth Factor
PMNs	Polymorphonuclear Neutrophils
PRRs	Pattern Recognition Receptors
RAGE	Receptor for Advanced Glycation End Products
RIG-I	Retinoic Acid-Inducible Gene-I
RLRs	Retinoic Acid-Inducible Gene-I Like Receptors
ROS	Reactive Oxygen Species
S100s	S100 Proteins
SOFA	Sequential Organ Failure Assessment
STING	Stimulator of Interferon Genes
TAZ	Transcriptional Coactivator with PDZ-Binding Motif
TBK1	TANK-Binding Kinase 1
TLRs	Toll-Like Receptors
TNF-α	Tumor Necrosis Factor-α
TREMs	Triggering Receptors Expressed on Myeloid Cells
TRP	Transient Receptor Potential
UDPG	Uridine Diphosphate Glucose
VEGF	Vascular Endothelial Growth Factor
VEGFR	Vascular Endothelial Growth Factor Receptor
